# The Effect of High Altitude on Short-Term Outcomes of Post-hemorrhoidectomy

**DOI:** 10.7759/cureus.33873

**Published:** 2023-01-17

**Authors:** Abdullah Al-Sawat, Noor Fayoumi, Mohammed A Alosaimi, Abdulaziz S Alhamyani, Albaraa M Aljuaid, Abdulelah M Alnefaie, Khalid F Alhejji, Muhammad Z Ali

**Affiliations:** 1 Department of Surgery, College of Medicine, Taif University, Taif, SAU; 2 Department of General Surgery, King Abdulaziz Specialist Hospital, Taif, SAU; 3 College of Medicine, Taif University, Taif, SAU; 4 Department of General Surgery, Alhada Armed Forces Hospital, Taif, SAU

**Keywords:** saudi arabia, high altitude, complications, short-term outcomes, post-hemorrhoidectomy

## Abstract

Background

Hemorrhoids are caused by the distal displacement of the hemorrhoidal cushions and venous distention. Hemorrhoidal illness is still a prevalent issue. Hemorrhoidal symptoms affect 5% of the general population and 50% of people over the age of 50. Surgical therapy ensures satisfactory results that are much better than those obtained with conservative treatments, particularly for grade III and IV hemorrhoids.

Objectives

This study aims to compare postoperative complications of hemorrhoidectomy among patients who live in a high-altitude region (Taif) and a sea-level (low-altitude level) region (Jeddah).

Methods

This retrospective study was conducted among patients who underwent hemorrhoidectomy between January 2019 and January 2022 in Taif and Jeddah, Saudi Arabia. Simple random sampling was used to select the study population, and data were collected from patient files. Data analysis was conducted using IBM SPSS Statistics for Windows, version 23.0 (IBM Corp., Armonk, NY).

Results

A total of 135 patients were included in this study. Most of our patients were males (73.3%). Patients from Jeddah represented more than half of the study population, and 45.2% were from Taif. The majority of low-altitude area patients require less than a week to return to normal activities (54.1%), whereas the majority of high-altitude area patients (59%) require more than a week (P = 0.047). 94.1% of participants felt pain on Day 1, and 54.1% had pain on Day 7. According to our findings, approximately one-fourth of patients (25.2%) developed recurrent hemorrhoids, and 27.4% experienced recurrent hemorrhoid symptoms. When we compared low-altitude regions and high-altitude regions in postoperative complications of hemorrhoidectomy, we found that urinary retention was most common in the high-altitude regions (37.7%).

Conclusion

Our results found that urinary retention was the most common complication in the high-altitude region. Early return to regular activity with less postoperative pain on Day 7 was a significant finding among low-altitude area patients.

## Introduction

Hemorrhoids are also known as piles. It is a common problem that affects people between 45 and 60 [1,2]. It is a swollen or distended vein in the lower part of the rectum (internal hemorrhoids) or beneath the skin around the anus (external hemorrhoids) [3], which may occur because of the weakening of the surrounding structures that support hemorrhoids along with the increased intra-abdominal pressure that comes with pregnancy, obesity, or excessive straining during bowel movements [4,5]. The patient would suffer from pain, itching, bleeding, or even prolapse (bulging out of the anus) [1,2]. The majority of cases only need conservative treatment like eating more fiber, some kind of exercise, and over-the-counter creams [6]. Only 10% of patients with recurrent bleeding or large prolapse require surgical intervention [7]. The gold standard treatment for these patients is hemorrhoidectomy, which provides fast and long-lasting results [8], but this type of surgery is not without complications, which although usually short-term and non-life-threatening, may last about one week only, in most cases [2]. The most prevalent postoperative complication that comes along is pain, which most likely happens because of a spasm of the internal sphincter [9]. Some studies have mentioned that local sepsis has a role in pain, as the administration of metronidazole in the first week helps relieve the pain [10,11]. Mild bleeding tends to occur in the first 24-48 hours after surgery; it usually happens because of poor intraoperative hemostasis [12]. Urinary retention has been reported to be around 34% in patients after hemorrhoidectomy [13]. Two percent to 10% of patients would report fecal incontinence due to accidental sphincter injury, which causes a great impact on quality of life [14]. Sporadic cases (about 5%) have been reported as transient bacteremia [15].

There are quite a lot of studies that mention the effect of oxygen after surgeries, as oxygen is an important factor that affects wound healing significantly in the inflammatory and proliferative phases [16]. It enhances the inflammatory phase by stimulating neutrophils to produce a large amount of reactive oxygen species (ROS), which helps kill microorganisms [17,18], and there is clinical evidence [19] from a trial that was conducted on 300 colorectal surgery patients; some of them received 30% postoperative oxygen, and the others received 80% postoperative oxygen. They found that the risk of surgical site infection was 39% lower in the ones who received 80% oxygen. A study also found that patients with a chronic injury who were treated with hyperbaric oxygen therapy had high levels of vascular endothelial growth factor (VEGF), which aids in angiogenesis [20]. As a result, this study aims to determine the prevalence of hemorrhoidectomy complications in high-altitude regions that experience relative hypoxia.

## Materials and methods

Study design

This study was a retrospective study conducted in Taif and Jeddah City, Kingdom of Saudi Arabia, between July 2022 and December 2022.

Study population and sampling methodology

This study had a sample size of 135 patients and the data were gathered at the King Abdulaziz Specialist Hospital and Alhada Armed Forces Hospital in Taif (high-altitude area) and the King Fahad Armed Forces Hospital in Jeddah (low-altitude area), both in Saudi Arabia. The inclusion criteria were patients over the age of 15 who had hemorrhoidectomy with confirmed short-term postoperative complications from date of January 2019 to January 2022. In addition, patients younger than 15 years or with missing clinical data within their medical files or not within the time period were excluded. 

Data analysis

Statistical analysis was done using IBM SPSS Statistics for Windows, version 23.0 (IBM Corp., Armonk, NY). The mean and standard deviation were reported for continuous variables while categorical variables like gender were described using frequencies and percentages. A chi-square test and Fisher’s exact test were used to compare categorical variables like gender. The p-value <0.05 was considered significant.

Ethical considerations

Ethical approval was provided by the Institutional Review Board (IRB) of Directorate of Health Affairs - Taif HAP-02-T-067-Approval Number 639, IRB of Alhada Armed Forces Hospital, Taif (REC.2022-609) and IRB of King Fahad Armed Forces Hospital, Jeddah (REC 512). It was performed in accordance with the Declaration of Helsinki and written informed consent was waived due to its retrospective nature.

## Results

Characteristics of the participants

This study included a total of 135 patients. The majority of them were males (73.3%), and less than one-third were females (26.7%). Patients from the low-altitude area (Jeddah) represented 54.8% of the study population, and patients from the high-altitude area represented 45.2%. Furthermore, we found that 45.2% of patients were between the ages of 18 and 40, with this age group accounting for the majority of patients living in low altitudes. Almost half of the patients from high-altitude areas were between the ages of 41 and 60. When we calculated the BMI of our patients, we found that more than half (59%) of those from high-altitude areas had a BMI of less than 25. In contrast, only 27% of patients living in low-altitude areas had a BMI of less than 25. This difference was found to be statistically significant (P-value = 0.001) (Table 1).

**Table 1:** Baseline characteristics of the participants (n=135)

Variable	Categories	Low-altitude area (n=74)	High-altitude area (n=61)		Total (n=135)	P-value
		n (%)				
Gender	Male	52 (70.3)		47 (77)	99 (73.3)	0.375
	Female	22 (29.7)		14 (23)	36 (26.7)	
Age (years)	18–40	34 (45.9)		27 (44.3)	61 (45.2)	0.343
	41–60	30 (40.5)		30 (49.2)	60 (44.4)	
	More than 60	10 (13.5)		4 (6.6)	14 (10.4)	
BMI (Kg/m^2^)	Less than 25	20 (27)		36 (59)	56 (41.5)	0.001
	25–30	27 (36.5)		12 (19.7)	39 (28.9)	
	More than 30	27 (36.5)		13 (21.3)	40 (29.6)	
Family history of hemorrhoid	Yes	33 (44.6)		21 (34.4)	54 (40)	0.230
	No	41 (55.4)		40 (65.6)	81 (60)	
Physical activity	Regular	23 (31.1)		32 (52.5)	55 (40.7)	0.012
	Not regular	51 (68.9)		29 (47.5)	80 (59.3)	
Smoking	Yes	30 (40.5)		23 (37.7)	53 (39.3)	0.737
	No	44 (59.5)		38 (62.3)	82 (60.7)	
Cough	Yes	12 (16.2)		17 (27.9)	29 (21.5)	0.101
	No	62 (83.8)		44 (72.1)	106 (78.5)	
Constipation	Yes	46 (62.2)		37 (60.7)	83 (61.5)	0.777
	No	28 (37.8)		23 (37.7)	51 (37.8)	
	Not applicable	0 (0)		1 (1.6)	1 (0.7)	
Heavy exercise	Yes	6 (8.1)		16 (26.2)	22 (16.3)	0.005
	No	68 (91.9)		45 (73.8)	113 (83.7)	

In terms of a family history of hemorrhoids, our findings revealed that the majority of the study population (60%) did not have a family history of hemorrhoids. In addition, our findings demonstrated that most patients from low-altitude areas admitted that their practice of physical activity was irregular. On the other hand, more than half of patients from high-altitude areas practiced regular physical activity, and this difference between both regions came to be significant (P-value = 0.012) (Table 1).

Additionally, our results showed that the majority of the study population were non-smokers (60.7%). The proportion of smokers was higher in low-altitude areas without any significant difference. Moreover, we found that the majority of patients did not suffer from cough (78.5%). On the other side, most of them had constipation (61.5%). Furthermore, performing heavy exercise was more common among patients from high-altitude areas. The vast majority of patients from low-altitude areas revealed that they had not performed heavy exercise (91.9%). This difference was found to be significant (P-value = 0.005) (Table 1).

Information about surgery for hemorrhoidectomy

Our results found that more than one-third of patients had grade III hemorrhoids, and about one-fourth of them had grade IV hemorrhoids; most of the patients from low-altitude areas had grade III hemorrhoids (P < 0.001). Moreover, our findings showed that 17% of patients had previous hemorrhoid surgery. The vast majority of patients had preoperative pain, and most of them received preoperative analgesia (83% and 65.2%, respectively). Only 5.9% of patients underwent reoperation.

In regards to the duration of surgery, we found that most patients spent less than one hour in surgery (77%), and most of them were from low-altitude areas, while only 20% spent more than one hour, and most of them were from high-altitude areas (P = 0.024). In addition, our results reported that more than half of the study patients lost less than 50 ml of blood during surgery, and most of them were from low-altitude areas (P = 0.006). The majority of patients (82.2%) stayed in the hospital for less than two days, and only 17.8% stayed for more than two days. Furthermore, 54.1% of patients reported that their first bowel movement began within 24 hours of surgery, and the majority of them were from high-altitude areas (P = 0.003) while 41.5% required more than 24 hours.

When assessing the recovery state of our patients, we reported that almost one-half of them needed more than a week to return to normal activities. The majority of low-altitude area patients (54.1%) required less than a week to return to normal activities, whereas the majority of high-altitude area patients (59%) required more than a week (P = 0.047). Ninety-four point one percent (94.1%) of participants felt pain on Day 1, and it was higher among patients from low-altitude areas (P = 0.001); 54.1% had pain on Day 7, and most of them were from high-altitude areas (P = 0.015). Our results found that about one-fourth of patients (25.2%) developed recurrent hemorrhoids, and 27.4% suffered from recurrent hemorrhoid symptoms (Table 2).

**Table 2:** Information about hemorrhoidectomy surgery Percentages were calculated within each column, * P-values were calculated using Fisher’s exact test, and other p-values by the chi-square test

Variable	Categories	Overall	Low-altitude area (n=74)	High-altitude area (n=61)	P-value
			n (%)		
Grade of hemorrhoids	I	10 (7.4%)	6 (8.1)	4 (6.6)	< 0.001
	II	14 (10.4%)	6 (8.1)	8 (13.1)	
	III	53 (39.3%)	37 (50)	16 (26.2)	
	IV	34 (25.2%)	21 (28.4)	13 (21.3)	
	Not applicable	24 (17.8%)	4 (5.4)	20 (32.8)	
Previous hemorrhoid surgery	Yes	23 (17%)	15 (20.3)	8 (13.1)	0.271
	No	112 (83%)	59 (79.7)	53 (86.9)	
Preoperative pain	Yes	113 (83.7%)	66 (89.2)	47 (77)	0.057
	No	22 (16.3%)	8 (10.8)	14 (23)	
Preoperative analgesia	Yes	88 (65.2%)	52 (70.3)	36 (59)	0.233*
	No	46 (34.1%)	22 (29.7)	24 (39.3)	
	Not applicable	1 (0.7%)	0 (0)	1 (1.6)	
Reoperation	Yes	8 (5.9%)	5 (6.8)	3 (4.9)	0.729*
	No	127 (94.1%)	69 (93.2)	58 (95.1)	
Duration of surgery	< 1 hour	104 (77 %)	62 (83.8)	42 (68.9)	0.024*
	> 1 hour	27 (20%)	12 (16.2)	15 (24.6)	
	Not applicant	4 (3%)	0 (0)	4 (6.6)	
Operative blood loss	< 50 ml	79 (58.5%)	51 (68.9)	28 (45.9)	0.006*
	> 50 ml	4 (3%)	3 (4.1)	1 (1.6)	
	Not applicable	52 (38.5%)	20 (27)	32 (52.5)	
Length of hospital stay	< 2 days	111 (82.2%)	62 (83.8)	49 (80.3)	0.644*
	> 2 days	23 (17%)	12 (16.2)	11 (18)	
	Not applicant	1 (0.7%)	0 (0)	1 (1.6)	
Time to first bowel movement	< 24 hours	73 (54.1%)	37 (50)	36 (59)	0.003*
	> 24 hours	56 (41.5%)	37 (50)	19 (31.1)	
	Not applicant	6 (4.4%)	0 (0)	6 (9.8)	
Time to normal activities	Less than a week	63 (46.7%)	40 (54.1)	23 (37.7)	0.047*
	more than a week	70 (51.9%)	34 (45.9)	36 (59)	
	Not applicant	2 (1.5%)	0 (0)	2 (3.3)	
Pain on day 1	Yes	127 (94.1%)	74 (100)	53 (86.9)	0.001*
	No	8 (5.9%)	0 (0)	8 (13.1)	
Pain on day 7	Yes	73 (54.1%)	33 (44.6)	40 (65.6)	0.015
	No	62 (45.9%)	41 (55.4)	21 (34.4)	
Recurrent hemorrhoids	Yes	34 (25.2%)	15 (20.3)	19 (31.1)	0.069*
	No	99 (73.3%)	59 (79.7)	40 (65.6)	
	Not applicant	2 (1.5%)	0 (0)	2 (3.3)	
Recurrent hemorrhoid symptoms	Yes	37 (27.4 %)	18 (24.3)	19 (31.1)	0.395*
	No	96 (71.1%)	54 (73)	42 (68.9)	
	Not applicant	2 (1.5%)	2 (2.7)	0(0)	

When we compared postoperative complications of hemorrhoidectomy in low-altitude and high-altitude regions, we found that urinary retention was most common in the high-altitude region (37.7%). Only 18.9% of patients from low-altitude regions developed urinary retention. This difference was found to be significant (P-value = 0.015). Bleeding was the most common postoperative complication overall, and other complications did not reveal any significant difference between the two regions, as shown in Table 3.

**Table 3:** Comparison between low-altitude regions (Jeddah) and high-altitude regions (Taif) in the postoperative complications of hemorrhoidectomy Percentages were calculated within each column, * P-values were calculated using Fisher’s exact test, and other p-values by the chi-square test

Post-operative complications	Sea level region (Jeddah)	High region (Taif)	Overall	P-value
	n (%)			
Bleeding	30 (40.5)	24 (39.3)	54 (40)	0.888
Need for blood transfusion	4 (5.4)	5 (8.2)	9 (6.7)	0.731*
Urinary retention	14 (18.9)	23 (37.7)	37 (27.4)	0.015
Residual hemorrhoidal tissue	15 (20.3)	9 (14.8)	24 (17.8)	0.404
Surgery for complication	2 (2.7)	5 (8.2)	7 (5.2)	0.244*
Anal stenosis	7 (9.5)	9 (14.8)	16 (11.9)	0.344
Anal fistula	4 (5.4)	4 (6.6)	8 (5.9)	1.000*
Anal fissure	6 (8.1)	6 (9.8)	12 (8.9)	0.726
Wound discharge	17 (23)	8 (13.1)	25 (18.5)	0.142
Other	2 (2.7)	4 (6.6)	6 (4.4)	0.409*

**Figure 1:**
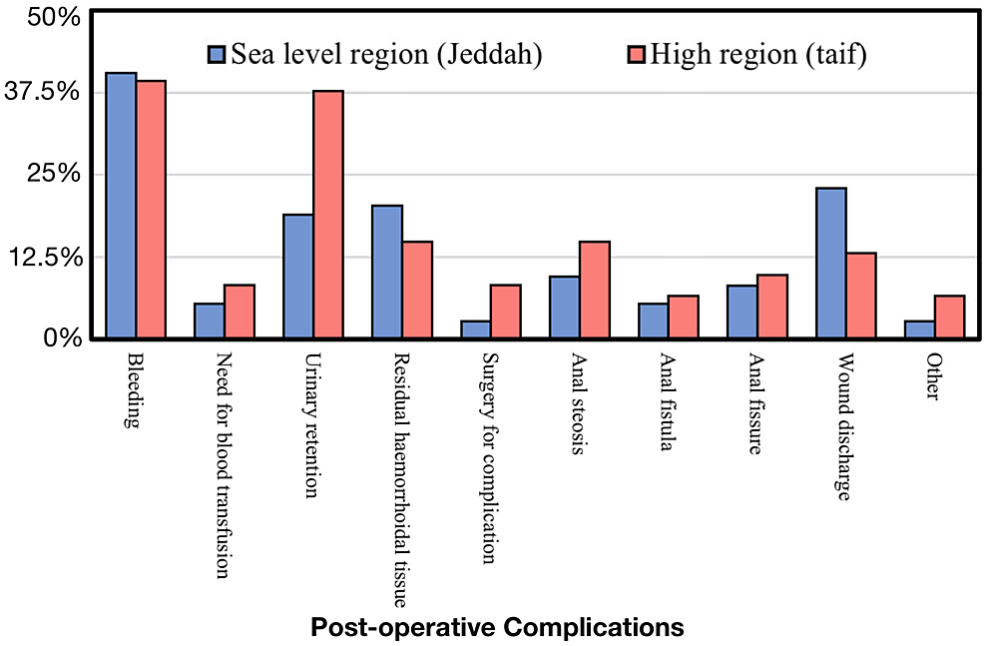
Postoperative complications between the high-altitude and low-altitude areas

Figure 1 shows the percentage of postoperative complications between the high-altitude and low-altitude areas.

## Discussion

The present study aimed to compare postoperative hemorrhoidectomy complications between patients living in high-altitude (Taif) and sea-level (Jeddah) regions. According to the severity of the condition, therapeutic treatment for hemorrhoids is rather well-established. Conservative treatment is generally recommended for grades I and II while surgical treatment is recommended for grades III and IV [21,22]. Although hemorrhoids are not fatal, the physical and psychological discomfort associated with numerous hemorrhoidal symptoms, such as anal bleeding, pain, and an itchy feeling, can have a significant impact on a person’s quality of life (QOL) [23].

Our results found that more than half of the patients from high-altitude areas had a BMI of less than 25. On the other hand, only 27% of patients from low-altitude areas had a BMI of lower than 25. This difference was found to be statistically significant (P-value = 0.001). Increased intra-abdominal pressure in an obese person with excessive body fat and visceral fat is hypothesized to cause venous congestion of the distal rectum and, as a result, contribute to hemorrhoid formation [24,25]. An earlier Korean study found that obesity and abdominal obesity were linked to an increased incidence of hemorrhoids [26]. Another study, however, found inconclusive results, revealing that being neither overweight nor obese was connected with the presence of hemorrhoids [27].

Additionally, most of our patients had constipation (61.5%). This result is in line with another study conducted in the US, which reported that constipation was associated with an increased prevalence of hemorrhoids [27]. In addition, our findings demonstrated that most patients from low-altitude areas admitted that their practice of physical activity was irregular. On the other hand, more than half of patients from high-altitude areas practiced regular physical activity, and this difference between both regions came to be significant (P-value = 0.012). Previous research found that sedentary behavior, but not physical exercise, was related to lower risk (OR 0.80, 95% CI 0.65-0.98) [27]. Another study found that for women, no regular walking was associated with a higher incidence of hemorrhoids (OR, 1.11; 95% CI, 1.00 to 1.23 and OR, 1.62; 95% CI, 1.17 to 2.25, respectively) [26].

Our results found that more than one-third of patients were diagnosed with grade III hemorrhoids, and about one-fourth of them had grade IV hemorrhoids. This is confirmed by another study in Saudi Arabia, which showed that 86% of patients had grade III hemorrhoids, and four (14%) had grade IV hemorrhoids [28]. In addition, we found that the vast majority of patients had preoperative pain, and most of them received preoperative analgesia (83% and 65.2%, respectively). This was consistent with another study conducted in Saudi Arabia, which showed that postoperative pain was tolerable (non-persistent) in 28 (93%) cases, whereas two (7%) experienced mild pain requiring additional analgesia [28]. Previous research concluded that the following recommendations are supported by the literature in order to reduce pain associated with hemorrhoids surgery: local anesthetic infiltration, either as a sole technique or as an adjunct to general or regional anesthesia; combinations of analgesics (non-steroidal anti-inflammatory drugs, paracetamol, and opiates); and a stapled operation [29]. Most of the respondents spent less than one hour in surgery, and only 20% spent more than one hour. Another study showed similar results [28].

Regarding the surgery compilation, we found that bleeding was the most common postoperative complication overall. The majority of bleeding complications occur during surgery at the staple line and should be handled with suturing of the bleeding spots following a careful assessment of the stapled suture line [30]. According to another study, 1.5% of participants suffered bleeding. Of these, 0.8% of patients had stump bleeding while 0.7% had marginal bleeding [31]. Furthermore, we found that urinary retention was most common in high-altitude regions (37.7%). Only 18.9% of respondents from low-altitude regions developed urinary retention. This is supported by another study in Saudi Arabia, which stated that urinary retention was the most common complication found in 16% of patients [28]. Several studies estimated that the incidence of urinary retention ranges from 0% to 34% and from 0% to 22% after stapled hemorrhoidopexy [32,33].

One of the important limitations of this study was the small sample size, which limited the generalizability of our results.

## Conclusions

Our results found that urinary retention was the most common complication in high-altitude regions. Early wound healing and high oxygen levels may help patients from low-altitude areas return to regular activity more quickly. On the other hand, low oxygenation and delayed wound healing might explain why pain on Day 7 is more severe in patients with low altitude. We recommend conducting multiple studies and clinical trials to evaluate the effect of high altitude on the short-term outcomes of post-hemorrhoidectomy. Careful patient education allows surgical hemorrhoidectomy to be performed with a very low incidence of complications.
